# Notes and News

**Published:** 1901-08-03

**Authors:** 


### Notes and News.

The two patients removed off the *Ormuz* last week are now certified not to be suffering from the plague.

Abergavenny is to have a new hospital, the foundation stone of which has just been laid by the Marquess of Abergavenny.

Mr. J. Pierrepont Morgan has given upwards of £200,000 for the purpose of erecting three buildings for the Harvard Medical School.

Owing to an absence of male applicants, the Birkenhead Board of Guardians have appointed a lady as house surgeon to their workhouse.

The unusual function of laying two foundation stones for one establishment has been held at Bury as a commencement of the new infectious diseases hospital, the chairman and vice-chairman each laying a stone. Forty-seven architects competed, three prizes being offered for the best plans. The first prize was won by Messrs. Pole and Little, of London. Accommodation will be provided for 21 scarlet fever, 15 typhoid, 6 diphtheria, and 8 isolation cases.

It is interesting to note that a lady has been elected Chairman of the Board of Management of the Swansea Hospital. Miss Dillwyn, the lady in question, had a pleasant duty on occupying the chair for the first time at the annual meeting, as she was able to announce that the anonymous donor of £5,000 towards the establishment of a convalescent home for the hospital was prepared to double the amount of the gift, leaving only £2,000 to be subscribed. Miss Dillwyn offered to subscribe £100 herself with the hope that nine other donors of the same amount would come forward.

Medical men interested in French watering places may make a pleasant little tour by joining the excursion organised by Dr. Carron de la Carrière, of Paris, under the title of "Voyage d'Étude Medicale. The tourists, who must be medical men, or the wives of medical men, assemble at Uriage on August 31st or September 1st, and after visiting a large number of watering places in the Dauphiné and in Savoy, separate at Evian on September 12th. Between the frontier and places of meeting and separation the railway companies charge half fares, and the total cost while with the party is 300 francs.

The Winsley Sanatorium for Consumption, which is to be erected for the use of Wiltshire and Somerset, will be situated on a fine site overlooking the Wiltshire Downs. The building will consist of a main building with wings at both ends, each containing three storeys, accommodation being provided for sixty patients. The architects are Messrs. Silcock and Reay, of Bath.

Last month a new operating theatre was opened by Mr. Edmund Owen at the Cardiff Infirmary. It is placed contiguous to the accident entrance, and is provided with entrance lobby, anæsthetic room, and doctor's room. The lights are on the northern side, the whole of the roof being of glass. Ventilation is secured by a powerful motor fan. A new sterilising apparatus has been added.

After nearly fifty years the old Newport Infirmary has been sold, and during the present week the patients have been removed to the new Newport and Monmouthshire Hospital, which has been erected at a cost of £40,000, upon a sight given by Lord Tredegar. The new hospital largely owes its existence to Dr. Garrod Thomas, since it was his offer of £5,000 towards a new hospital which first set the scheme afoot.

An adjourned meeting of members of the medical profession practising obstetrics and gynæcology was held in London on July 24th, under the presidency of Sir John Williams, at which it was unanimously resolved to establish a new journal, to be called "The British and Colonial Journal of Obstetrics and Gynæcology," and to raise the funds required for its publication by the formation of a limited liability company.

The David Lewis Trust is about to be utilised to establish another splendid work for the benefit of the public. This is to be an epileptic colony in Cheshire for 200 inmates. Mr. B. Levy, Mr. David Lewis's legatee, has caused a scheme to be prepared, based upon the lines of similar institutions on the Continent. The establishment will cost about £120,000, and an appeal will be made for the endowment of the colony. Through the energy of some Manchester gentlemen, with whom the idea originated, about £8,000 has already been collected.

The success of the "open-air" treatment at the North London Hospital for Consumption, Mount Vernon, Hampstead, is well known. For some months past the number of applications for admission has greatly exceeded the accommodation at the disposal of the Committee, and with a view of extending the useful work which the hospital is doing, a generous friend, who desires to remain anonymous, has placed a sum of money at the disposal of the Committee to erect a country branch and convalescent home to contain 100 beds to which patients can be transferred from the hospital. In addition to the purchase money, a sum of £1,000 per annum has been promised towards the endowment of this branch by the same friend, whose munificent gift will exceed £100,000. The Committee have already purchased 60 acres of land, some 350 feet above sea-level, lying on sand and gravel, well sheltered by woods and trees and situated on the borders of Hertfordshire, and the erection of the building will be proceeded with forthwith. The Committee trust that the generous support of the public will be forthcoming in order that the balance of additional income required (some £4,000) may be subscribed.

